# New identification and significance of Early Cretaceous mafic rocks in the interior South China Block

**DOI:** 10.1038/s41598-021-91045-1

**Published:** 2021-05-31

**Authors:** Hui-Min Su, Shao-Yong Jiang, Jia-Bin Shao, Dong-Yang Zhang, Xiang-Ke Wu, Xi-Qiang Huang

**Affiliations:** 1 State Key Laboratory of Geological Processes and Mineral Resources, Collaborative Innovation Center for Exploration of Strategic Mineral Resources, School of Earth Resources, China University of Geosciences, Wuhan, 430074 China; 2 Guangxi Institute of Geological Survey, Guangxi Bureau of Geology and Mineral Prospecting and Exploitation, Nanning, 530023 China

**Keywords:** Solid Earth sciences, Geochemistry, Petrology

## Abstract

Early Cretaceous mafic rocks are first reported in the northern Guangxi region from the western Qin-Hang belt in the interior South China Block. A systematic investigation of zircon U–Pb dating, whole-rock geochemistry, Sm–Nd isotopes and zircon Hf–O isotopes for these mafic rocks reveals their petrogenesis and the mantle composition as well as a new window to reconstruct lithospheric evolution in interior South China Block during Late Mesozoic. Zircon U–Pb dating yielded ages of 131 ± 2 Ma to 136 ± 2 Ma for diabase and gabbro from Baotan area, indicating the first data for Early Cretaceous mafic magmatism in the western Qing-Hang belt. These mafic rocks show calc-alkaline compositions, arc-like trace element distribution patterns, low zircon ε_Hf_(*t*) of − 9.45 to − 6.17 and high δ^18^O values of + 5.72 to + 8.09‰, as well as low whole-rock ε_Nd_(*t*) values of − 14.27 to − 9.53. These data suggest that the studied mafic rocks are derived from an ancient lithospheric mantle source that was metasomatized during Neoproterozoic subduction. Thus, the occurrence of these mafic rocks indicates a reactivation of Neoproterozoic subducted materials during an extension setting at Late Mesozoic in the western Qin-Hang belt, an old suture zone that amalgamates the Yangtze and Cathaysia blocks.

## Introduction

**Figure 1:**
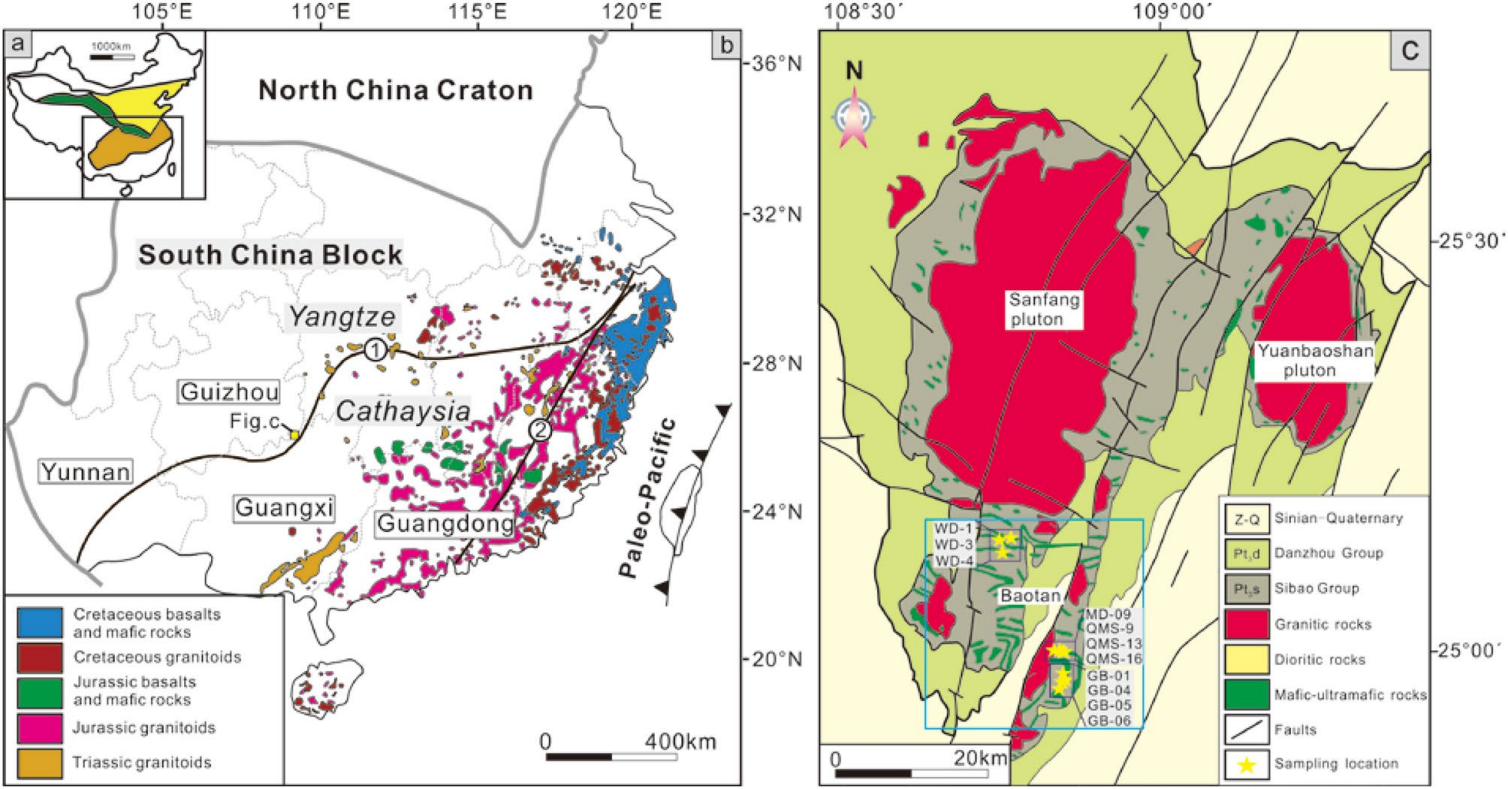
(**a**) Major tectonic units of China (modified from Ref.^5^). (**b**) Simplified map showing the distributions of the Mesozoic magmatism in SCB (modified from Ref.^5^). ① Qinzhou-Hangzhou belt, ② Zhenghe-Dapu fault zone. (**c**) Geological map of the northern Guangxi region showing the distribution of granites and mafic–ultramafic rocks with sample locations (modified from Ref.^6^).

South China Block (SCB) can be divided into the Yangtze Block (YB) and Cathaysia Block (CB) separated by the Qin-Hang belt (Fig. 1a,b), which was suggested as a Paleo-subduction zone in the Late Mesoproterozoic to Early Neoproterozoic, and afterwards the SCB underwent multiple episodes of intracontinental reworking^1^. In the CB, occurrences of Late Mesozoic mafic rocks and basalts are widely reported in particular in the eastern part of CB which are thought to be related to paleo-Pacific plate subduction^2^. In the Qin-Hang belt (QHB), occurrence of Early-Middle Jurassic mafic intrusive and volcanic rocks are reported only in the Nanling Range and eastern QHB, such as the Daoxian and Ningyuan basalts from southern Hunan (150–154 Ma and 170–174 Ma)^3^, and Antang basalts from central Jiangxi (168 Ma)^4^, however, up to now, no Late Mesozoic mafic rocks have been reported in the western QHB which locates further interior in the SCB. Therefore, it is still highly debatable whether or not the paleo-Pacific plate subduction can reach up to the interior SCB as far as the YB, and if so, what is the role of the paleo-suture zone of Neoproterozoic that amalgamates the two blocks of YB and CB. In this paper, we report our recent investigations that identified several Late Mesozoic mafic intrusions (gabbro and diabase) from the Baotan area of the northern Guangxi region which located along the western QHB in the YB side just next to the western interior CB (Fig. 1b,c). Our new data suggest that these mafic rocks are generated via reactivation of paleo-subduction derived materials during an extension setting at Late Mesozoic in the western QHB, an old suture zone that amalgamates the Yangtze and Cathaysia blocks.

## Geology of the studied region and occurrence of mafic rocks

In the northern Guangxi region of the western QHB occur mainly of Precambrian (meta-) sedimentary strata of Sibao Group and Danzhou Group and igneous rocks of early and middle Neoproterozoic, including voluminous granitoids and mafic–ultramafic rocks (Fig. 1c). The majority of the mafic–ultramafic intrusions are distributed in the Baotan, Sanfang and Yuanbaoshan areas (Fig. 1c) with more than 300 outcrops and a total exposed area of ca. 140 km^2^ that are dated at 812–855 Ma^7–9^.

Both gabbro and diabase occur as irregular shaped intrusions in the Mandong area that intruded into Neoproterozoic metasandstone strata (Supplementary Fig. S1a,b). The gabbro is also in fault contact with the Neoproterozoic mineralized pyroxenite (Supplementary Fig. S1c). The gabbro in Gaobang and diabase in Wende occur as stock and small intrusion that intruded into the Sibao Group (Supplementary Fig. S1d). Different from the widespread Neoproterozoic mafic rocks that have been subjected to variable degrees of alteration and/or mineralization in this region^10,11^, the mafic rock samples collected in this study are relatively fresh and suffered only very slightly alteration.

Mandong and Gaobang gabbros exhibit typical gabbro textures, mainly composed of 40% clinopyroxene, 50% plagioclase, 5% amphibole and 5% quartz (Supplementary Fig. S2a,b). Accessory phases mainly include apatite, titanite, and rutile. Mandong and Wende diabases are of porphyritic textures with phenocrysts of clinopyroxene, plagioclase and rare amphibole (Supplementary Fig. S2c,d). The matrix is microgranular and comprises dominant plagioclase, biotite, clinopyroxene, and minor K-feldspar and quartz. Minor clinopyroxene phenocrysts were locally replaced by an alteration assemblage of biotite (Supplementary Fig. S2d).

## Results

### Zircon U–Pb dating

**Figure 2:**
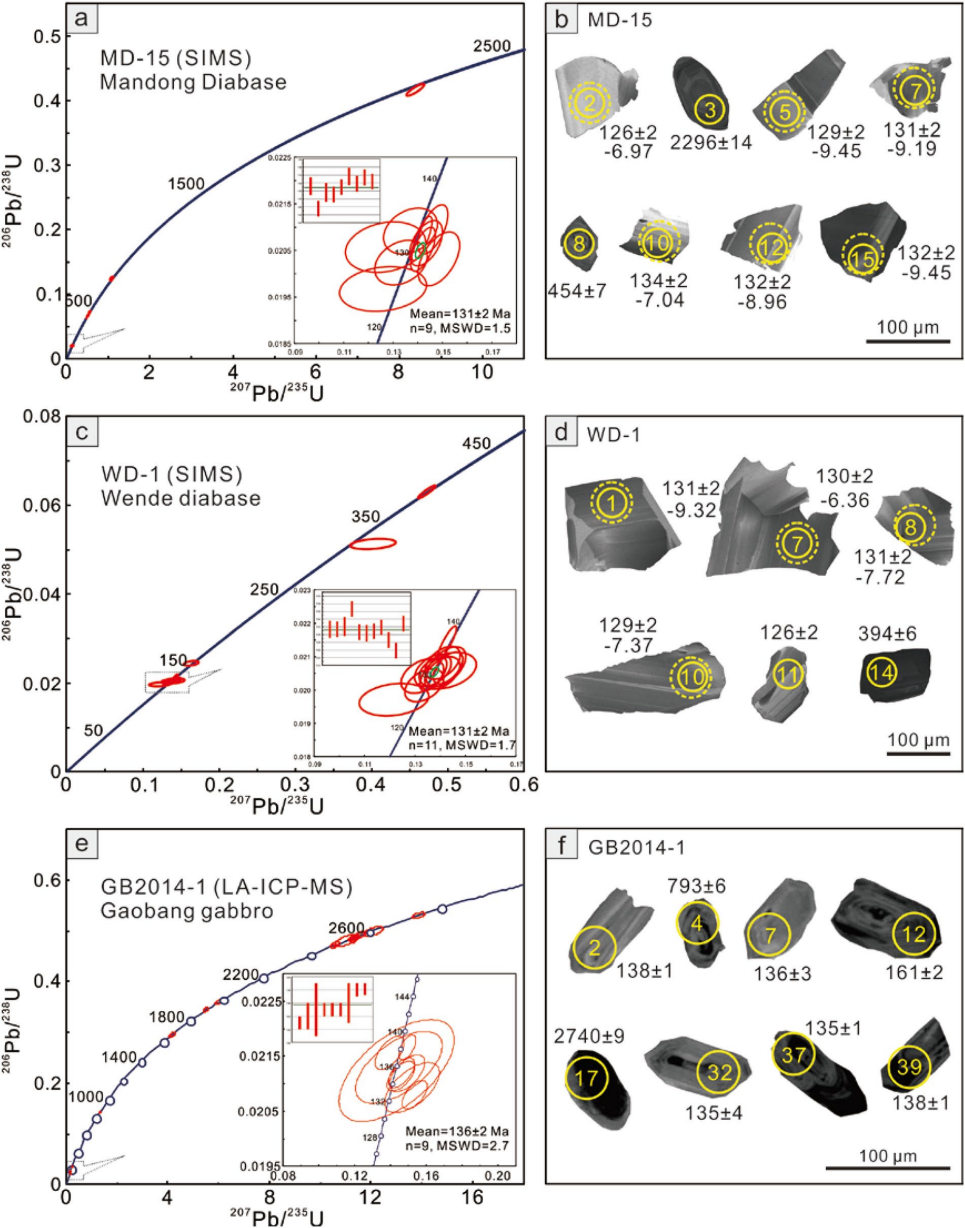
SIMS and LA-ICP-MS zircon U–Pb concordia diagrams for the studied mafic rocks (**a**,**c**,**e**) and cathodoluminescence (CL) images of the representative zircon grains for the studied mafic rocks (**b**,**d**,**f**). Solid circles represent the laser spots for U–Pb analysis, dashed spots for Hf isotope analysis.

Cathodoluminescence (CL) images of representative zircon grains from selected mafic rock samples in the northern Guangxi region are shown in Fig. 2. The Secondary ionization mass spectrometry (SIMS) and laser ablation inductively coupled plasma-mass spectrometry (LA-ICP-MS) zircon U–Pb dating results are listed in Supplementary Table S1 and shown in Fig. 2.

Fifteen analyses were carried out from Mandong (MD-15) and Wende (WD-1) diabase, respectively. An analysis point 9 on a whitish zircon from the sample MD-15 gives a youngest ^206^Pb/^238^U age of 81 ± 1 Ma, which may be caused by lead loss. Thus, this analysis was excluded from the final age calculation. Zircons from the Mandong diabase yield a weighted mean ^206^Pb/^238^U age of 131 ± 2 Ma (MSWD = 1.5, n = 9), those from the Wende diabase show a weighted mean ^206^Pb/^238^U age of 131 ± 2 Ma (MSWD = 1.7, n = 11). Older zircons (156–2246 Ma) (Fig. 2a,c) are also determined in both samples which indicate a detrital origin that originated from assimilation of wall rocks during magma ascent or inherited grains from the magma source region^12,13^. The Gaobang gabbro also show a wide range of zircon ages with nine youngest analyses forming a tight cluster and give a weighted mean ^206^Pb/^238^U age of 136 ± 2 Ma (MSWD = 2.7) (Fig. 2e). Therefore, the age data from the above studied samples indicate an early Cretaceous age for the mafic magmatism in northern Guangxi, which is the first report of Late Mesozoic mafic rocks in this region.

### Major and trace element concentrations

**Figure 3:**
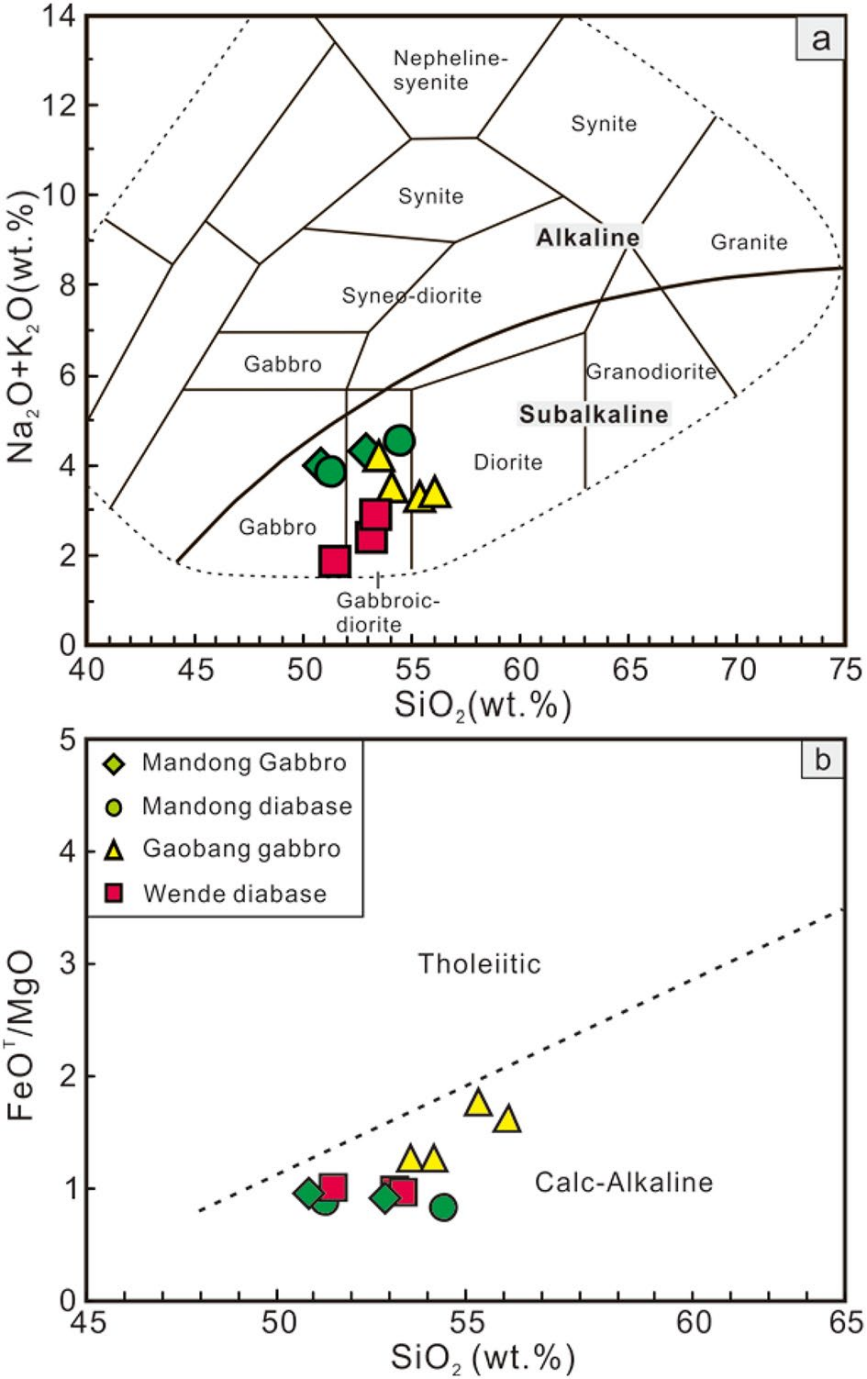
(**a**) Na_2_O + K_2_O versus SiO_2_ diagram and (**b**) FeO^T^/MgO versus SiO_2_ diagram for the Early Cretaceous mafic rocks in the western QHB.

The studied mafic rocks are characterized by elevated SiO_2_ (50.86–56.09 wt%), unusually low TiO_2_ (0.33–0.65 wt%) and moderate CaO (6.16–9.46 wt%) and Al_2_O_3_ contents (12.34–17.20 wt%). In addition, these samples show relatively high FeO^T^ (11.10–15.48 wt%) and variable MgO (4.99–9.81 wt%) concentrations with Mg^#^ value of 50–68 (Supplementary Table S2). Plot of Na_2_O + K_2_O vs. SiO_2_ shows that these samples belong to subalkalic gabbro and gabbroic-diorite (Fig. 3a). On the FeO^T^/MgO versus SiO_2_ diagram (Fig. 3b), all samples plot in the calc-alkaline field.

**Figure 4:**
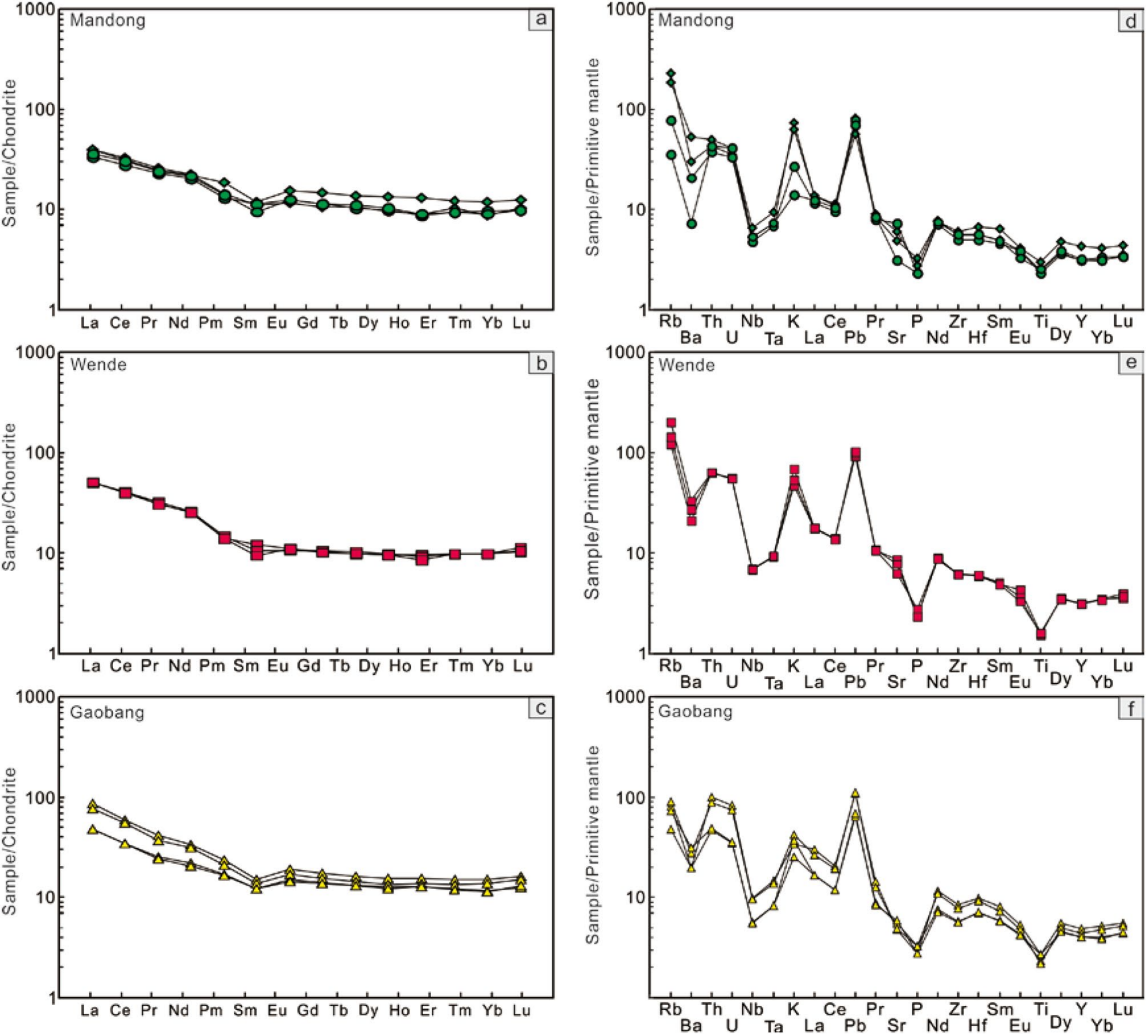
Chondrite-normalized REE patterns (**a**–**c**) and primitive mantle normalized multi-element variation diagrams (**d**–**f**) of the studied mafic rocks.

These mafic rocks have lower total rare earth element (REE) contents (49.0–96.1 ppm) (Fig. 4a–c). They display broadly similar REE pattern showing an enrichment in light rare earth elements (LREE) and flat heavy rare earth elements (HREE) pattern with (La/Yb)_n_ ratios of 3.3 to 5.7 and (Gd/Yb)_n_ ratios of 1.1–1.4 (Fig. 4a–c; Supplementary Table S2). A slight negative Eu anomaly (Eu/Eu* = 0.70–0.98) is present. On the primitive mantle-normalized multi-element diagram, the studied mafic rocks exhibit strong enrichments in Rb, Th, U and Pb and depletions in Nb, Ta, P and Ti (Fig. 4d–f), indicating a typical signature of continental arc basalt^14,15^.

### Nd-Hf–O isotopes

**Figure 5:**
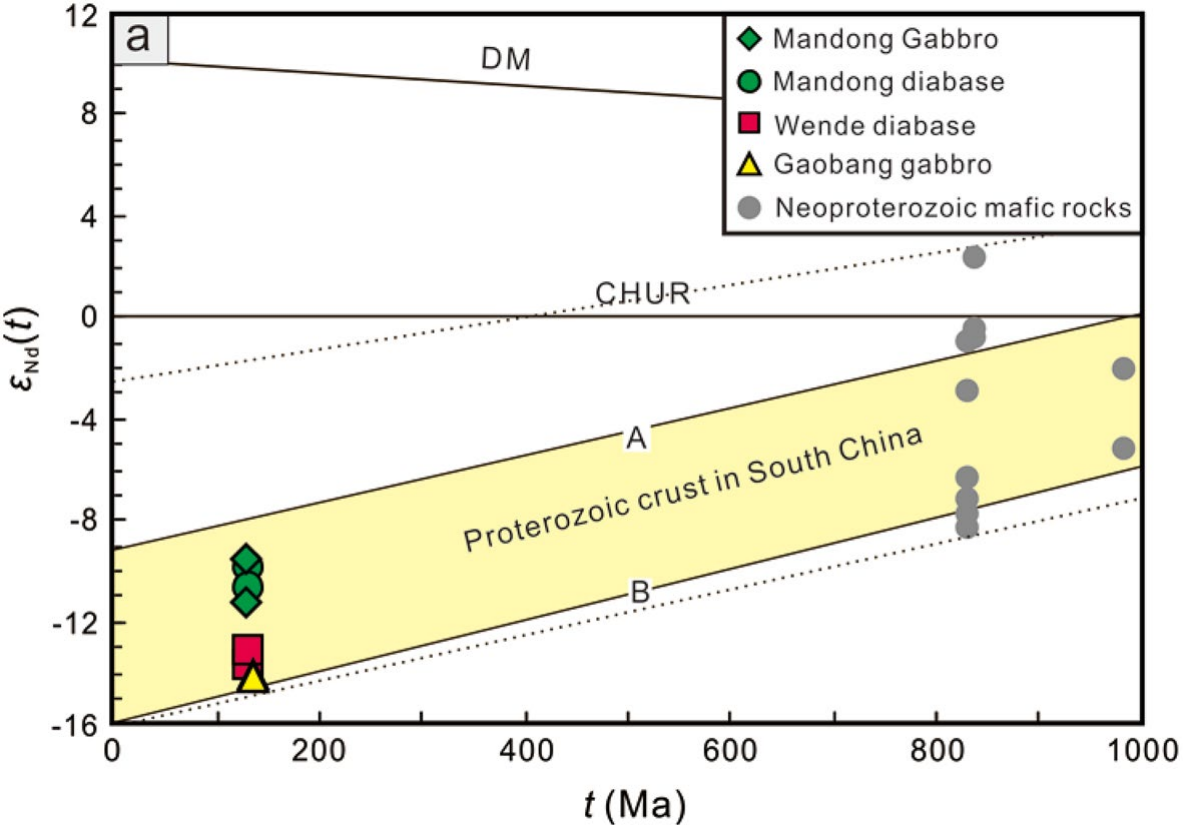
Plots of *ε*_Nd_(*t*) versus *t* for the studied mafic rocks in the Baotan area.

Mandong samples have ^147^Sm/^144^Nd ratios ranging from 0.1275 to 0.1683 and ^143^Nd/^144^Nd ratios from 0.51201 to 0.51213, corresponding to *ε*_Nd_(*t*) values of − 9.53 to − 11.23 (Fig. 5, Supplementary Table S3). The Wende diabases show relatively lower ^147^Sm/^144^Nd and ^143^Nd/^144^Nd ratios varying from 0.1092 to 0.1116 and 0.51187 to 0.51189, respectively, with lower *ε*_Nd_(*t*) values of − 13.65 to − 13.06 (Fig. 5, Supplementary Table S3). Gabbros from Gaobang, however, have the highest ^47^Sm/^144^Nd ratios of 0.1324 to 0.1374 and lowest ^143^Nd/^144^Nd ratios of 0.51186, corresponding to the lowest *ε*_Nd_(*t*) values of − 14.27 to − 14.16 (Supplementary Table S3).

**Figure 6:**
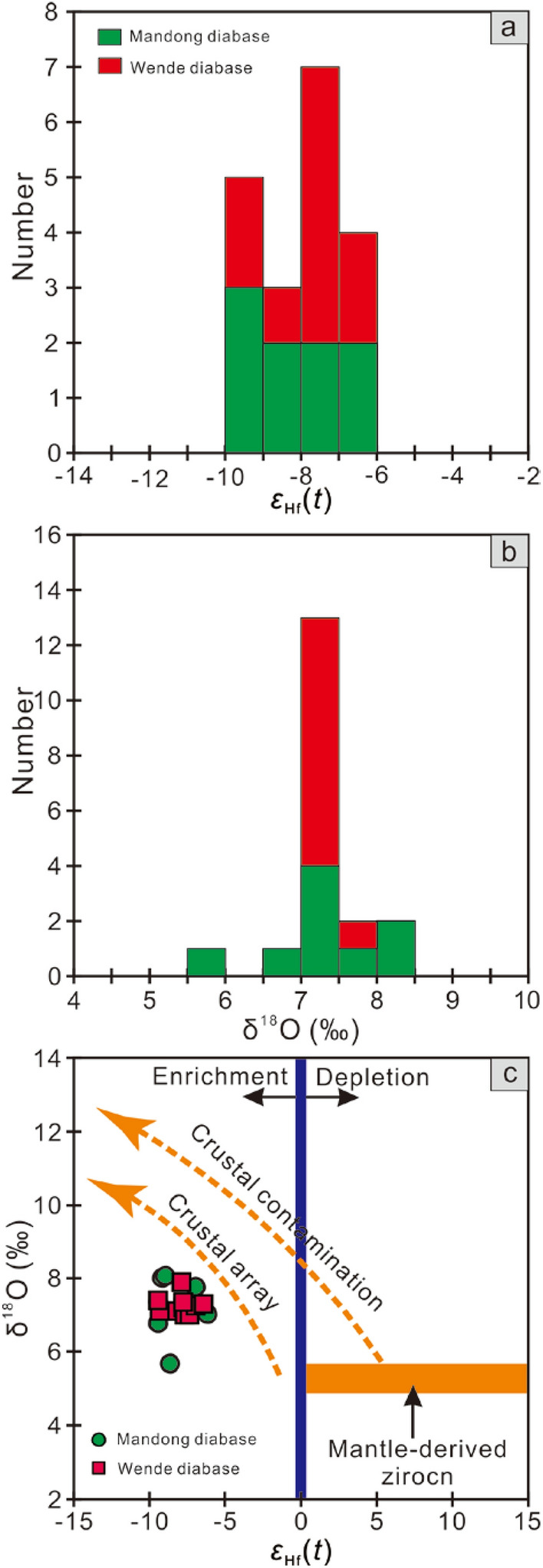
Histogram of Hf–O isotopic data (**a**,**b**) and *ε*_Hf_(*t*) versus δ^18^O correlation diagram (**c**) of zircon crystals from the studied mafic rocks in the Baotan area.

The Mandong and Wende samples have homogenous negative zircon *ε*_Hf_(*t*) values of − 9.45 to − 6.17 and − 9.39 to − 6.36, respectively (Fig. 6a, Supplementary Table S4). Furthermore, zircon grains from Wende samples have relatively homogenous O isotopic compositions, with δ^18^O values of + 7.03‰ to + 7.89‰, whereas, those from Mandong samples show variably larger range for zircon δ^18^O values from + 5.72‰ to + 8.09‰ (Fig. 6b, Supplementary Table S4). The oxygen isotope data in this study are significantly higher than the typical mantle value (+ 5.3 ± 0.3‰)^16^.

## Discussion

### New mafic magmatic event in the western Qin-Hang belt of interior South China Block

The mafic rocks in Baotan area from the Northern Guangxi region of the western QHB are traditionally thought to be contemporaneous with widely-exposed Neoproterozoic peraluminous granitoids^9,11^. Li et al.^7^ reported four mafic dykes that intruded into the Neoproterozoic Sibao Group with ages of 820–830 Ma (mean age 828 ± 7 Ma) by sensitive high-resolution ion microprobe (SHRIMP) zircon U–Pb method. Wang et al.^11^ conducted LA-ICP-MS zircon U–Pb dating for a layered diabase with an age of 812 ± 5 Ma. Weighted average ^206^Pb/^238^U ages of 855 ± 6 Ma and 835 ± 9 Ma for gabbros were obtained by LA-ICP-MS zircon U–Pb dating by Yao et al.^15^ and Chen et al.^9^, respectively. These ages indicate significant mafic magmatism at 855–812 Ma but also led to a widely-accepted assumption that the mafic rocks in the Northern Guangxi of the western QHB are all formed in Neoproterozoic period.

During our field work, we noticed some mafic intrusions which are much less weathered than those Neoproterozoic mafic rocks. Three samples from the intrusions were analyzed in different laboratories with different U–Pb dating methods. They gave consistent Early Cretaceous ages ranging from 136 ± 2 Ma to 131 ± 2 Ma (Fig. 2). Thus, we believe that these Early Cretaceous ages are reliable that may reveal a previous undiscovered mafic magmatism event in this region. We noticed that Wang et al.^17^ has reported a weighted average age of 100 ± 14 Ma for a lamprophyre in the adjacent region of Baotan in Northern Guangxi. Although this age is also Early Cretaceous, but the accurate data still need to be assessed because only two data points show ages of ~ 100 Ma among 30 analyses^17^. Therefore, our data have, for the first time, confirmed the presence of Early Cretaceous mafic rocks in the North Guangxi of the western QHB in spite of their small volume and unclear spatial distribution pattern.

### Crustal contamination and fractional crystallization of the mafic magma

**Figure 7:**
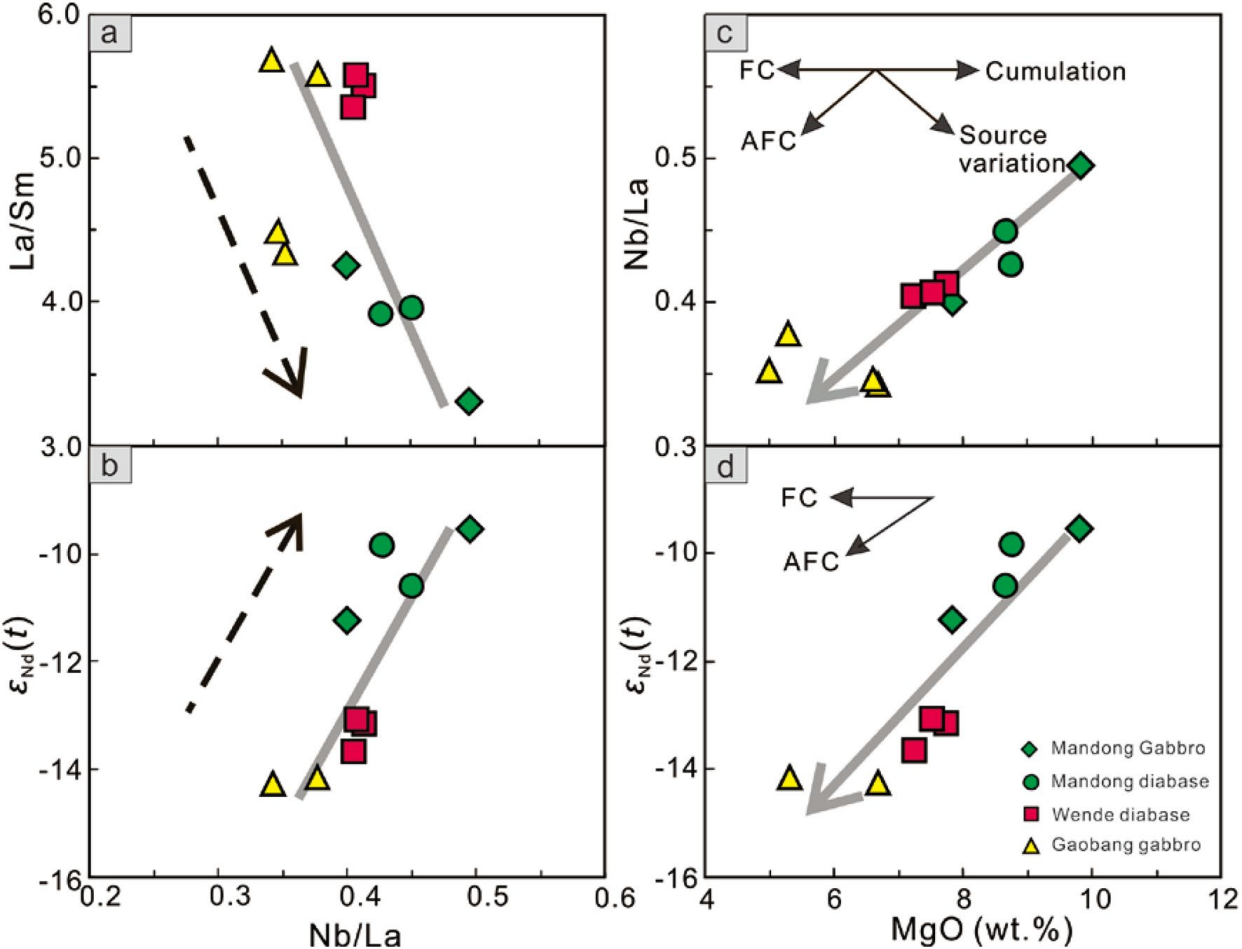
Plots of La/Sm and *ε*_Nd_(*t*) versus Nb/La (**a**,**b**), and Nb/La and *ε*_Nd_(*t*) versus MgO (**c**,**d**).

Although we observed the presence of some minor secondary minerals in some samples, most of the samples have relatively low loss on ignition (LOI) values of less than 3.0 wt%, and no obvious correlation is shown between the LOI and Nb/La and Th/La ratios, which suggests that the effect of post magmatic alteration is not significant for these studied samples. The petrographic evidence for zircon xenocrysts in the mafic rocks emphasizes the importance of crustal assimilation during magma ascent. In addition, on the *ε*_Nd_(*t*) versus *t* diagram (Fig. 5), the studied mafic rocks exhibit negative and variable *ε*_Nd_(*t*) values of − 14.27 to − 9.53 and plot in the Proterozoic SCB crust field. The considerable variation of negative *ε*_Nd_(*t*) values suggests effective contamination of the mafic rocks by crustal materials during magma emplacement. The effect of crustal contamination and assimilation fractionation crystallization (AFC) can be evaluated by correlations between major elements (such as MgO, SiO_2_ and FeO^T^) with trace element ratios (Nb/La, Nb/Th, Th/Ta and Zr/Nb) and isotope compositions^18–20^. Generally, La/Sm ratios decrease and *ε*_Nd_(*t*) values increase with increasing Nb/La ratios (Fig. 7a,b). In addition, clear positive correlations are found in the bivariation diagrams of Nb/La and *ε*_Nd_(*t*) versus MgO (Fig. 7c,d), similar to AFC trends. These lines of evidence indicate variable crustal contamination during their emplacement en route to the continental crust.

As presented in Fig. 6, the Wende mafic rocks show a relatively limited range of zircon *ε*_Hf_(*t*) values of − 9.36 to − 6.36 and δ^18^O values of + 7.03‰ to + 7.89‰. Although the Mandong samples exhibit relatively variable δ^18^O values from + 5.72‰ to + 8.09‰ for zircons, they have similar zircon *ε*_Hf_(*t*) values (− 9.45 to − 6.17) to the Wende samples. Furthermore, no covariation of Hf–O isotopes for the zircon grains from the Mandong and Wende mafic rocks could be observed (Fig. 6c). These facts imply that the effect of an AFC process on the Hf and O isotope composition of the zircon crystals is likely negligible.

**Figure 8:**
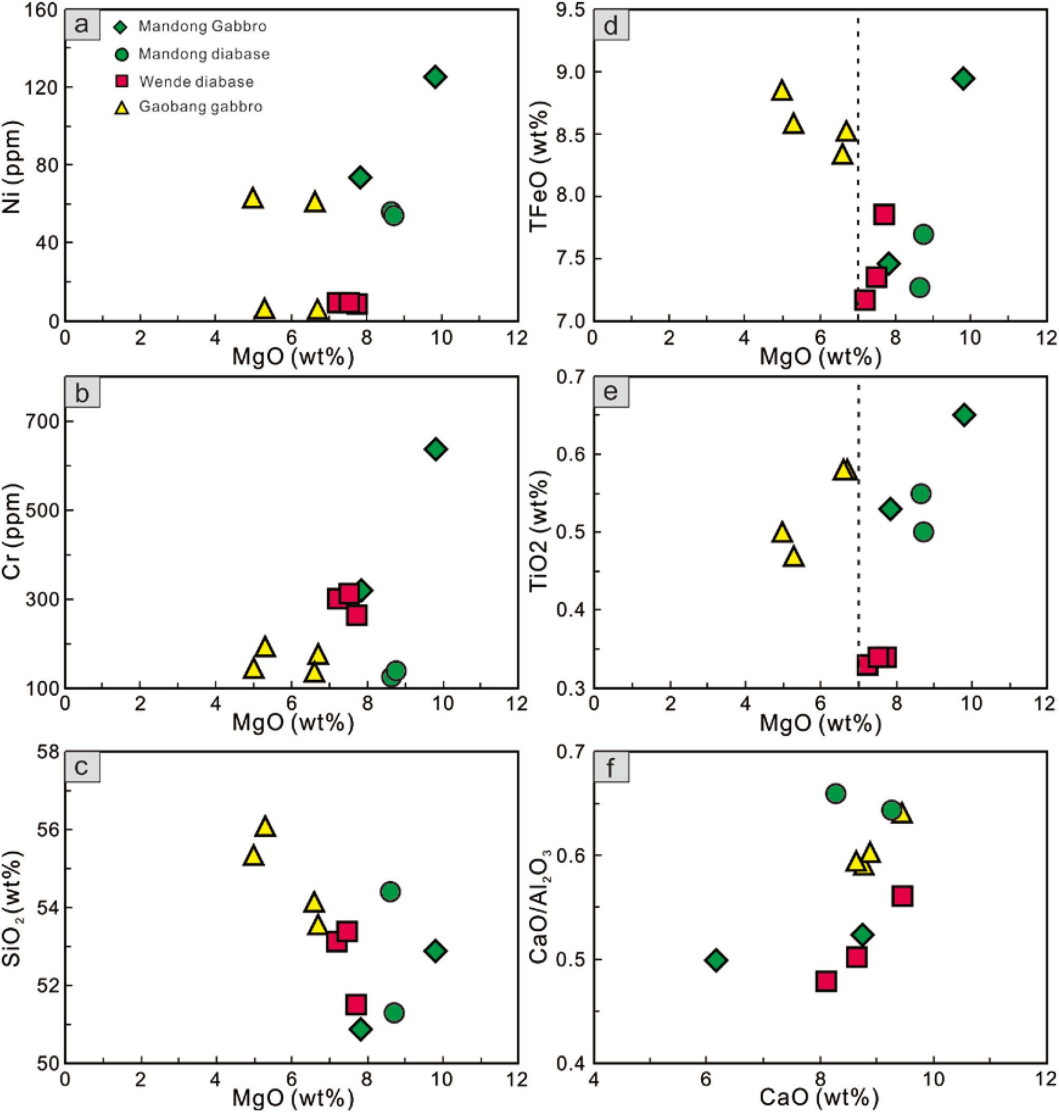
Variation diagrams of Ni, Cr, SiO_2_, TFeO and TiO_2_ versus MgO (**a**–**e**), and CaO/Al_2_O_3_ versus CaO (**f**).

Large variations in MgO (4.99–9.81 wt%) and compatible elements such as Cr (125–635 ppm) and Ni (5.98–125 ppm) indicate fractional crystallization of the parental magmas to varying degrees. Cr and Ni decrease sharply and SiO_2_ increases with decreasing MgO, showing significant fractionation of olivine and clinopyroxene (Fig. 8a–c). Rapid decreases in FeO^T^ and TiO_2_ with decreasing MgO at MgO > 7.0 wt% imply that Fe–Ti oxide were involved in the fractional phase at MgO > 7.0 wt% (Fig. 8d,e). The presence of a positive correlation between CaO/Al_2_O_3_ and CaO demonstrates that plagioclase fractionation is likely (Fig. 8f).

To minimize the effect of assimilation and fractionation crystallization, only the least contaminated and least evolved samples are chosen to constrain the source region. Among the mafic rocks in Baotan area, Mandong gabbros and diabases have the lowest SiO_2_ (50.86–54.40 wt%) and highest *ε*_Nd_(*t*) values (− 9.53 to − 11.23). Their Mg^#^ values range from 65 to 68 and therefore these samples are suggested to approximate to the compositions of primary melts.

### Magma source

**Figure 9:**
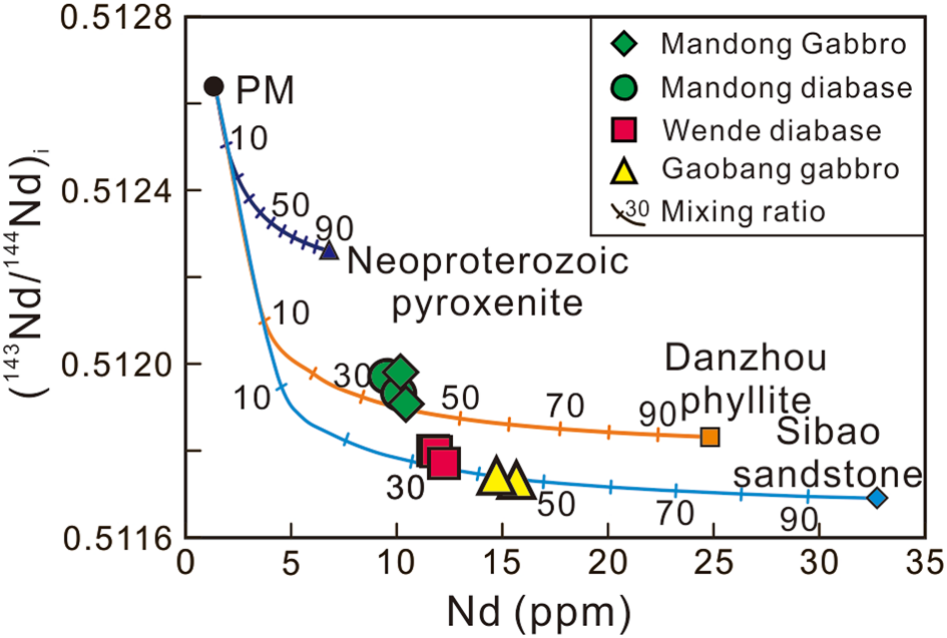
Whole rock (^143^Nd/^144^Nd)_i_ versus Nd (ppm) diagram showing the modeling results for crustal contamination for the Early Cretaceous mafic rocks.

All the mafic rocks in the Baotan area are characterized by relatively high MgO, Cr and Ni (up to 9.81 wt%, 635 ppm and 125 ppm, respectively), suggesting that these rocks originated from a lithospheric mantle source^21^. The crust-like trace element signatures, such as high LREE and large-ion lithophile elements (LILE) contents, low ratios of Nb/Ta (11.57–13.27), Ce/Pb (3.10–5.01) and Nb/U (3.99–5.53), indicate a great contribution of crustal materials in magma sources and/or during subsequent ascending processes^22–24^. We conducted quantitative modeling to evaluate the contribution of crustal materials based on Nd isotopic compositions by the (^143^Nd/^144^Nd)_i_-Nd plot (Fig. 9). The compositions of the Neoproterozoic metasedimentary rocks from the Sibao and Danzhou Group are taken as the crustal contaminant^25,26^. The simple modelling result suggests, that at least 30–45% crustal materials are required in the generation to achieve the observed Nd isotopic compositions of the studied mafic rocks (Fig. 9). However, the assimilation en route of such high-proportional crustal materials is obviously impossible for maintaining the mafic compositions. Moreover, the *ε*_Hf_(*t*) values of the zircon from these mafic rocks are negative and mostly range from − 9.47 to − 6.17, whereas their δ^18^O values in the range of + 5.72‰ to + 8.09‰, mostly higher than the mantle value (+ 5.3 ± 0.3‰)^16^. Thus, these zircon Hf–O isotopes are enriched and coherent with the Nd isotopic compositions of the least contaminated Mandong mafic rock samples. As mentioned above, the AFC process is unlikely responsible for the low *ε*_Hf_(*t*) values and higher δ^18^O for the zircon crystals in the mafic rocks. Thus, the observed isotopes abnormities of the zircons should be ultimately attributed to the composition of their parental magmas. Therefore, the crust-like geochemical signatures, such as high LREE and LILE contents and significant Nb–Ta–Ti negative anomalies, cannot totally be attributed to crustal contamination en route, and probably, are partially inherited from a mantle source modified by subduction-derived components in either recent or ancient metasomatism^27^.

Subduction is the major mechanism that carries enriched crustal components into mantle source. During the long evolutionary history of the SCB, the lithosphere mantle beneath it experienced several metasomatic events^28^. Among them, the Neoproterozoic subduction related to the collision between the Yangtze and Cathaysia blocks, or the so-called Jiangnan Orogeny, is the most important^29^.

In this study, we consider that the Neoproterozoic subduction event is most likely responsible for the crust-like components of the studied mafic rocks. Several lines of evidence support this proposal. Firstly, the antiquity of the metasomatic event would require the metasomatized domain to have remained chemically isolated for a long time, which would have allowed a general isotopic homogenization of the magma source(s)^30^. Whereas, the absence of isotopic homogenization within the source(s) usually corresponds to a relatively recent metasomatism^30^. Thus, the Hf isotopic homogeneity in the samples is more consistent with an ancient enrichment event rather than a relatively recent metasomatism in which highly isotopic variability is usually expected to be observed. Furthermore, there is a broad consensus that the Yangtze and Cathaysia blocks, following the northward subduction of an oceanic lithosphere beneath the southeastern Yangtze Block, amalgamated along the Jiangnan Orogen to form a united SCB in the Neoproterozoic time^31–33^. Subsequently, the SCB turned into an intracontinental tectonic development during the latest Neoproterozoic to Cretaceous period^34–36^. The uniform low *ε*_Hf_(*t*) values with Hf model ages ranging from 1.0 to 1.2 Ga also support a derivation from a Proterozoic metasomatized source. Finally, both the metasedimentary rocks within the Sibao Group and 860–830 Ma mafic rocks intruding into the lower Sibao Group exhibit arc-like geochemical affinity and these rocks were interpreted as the refractory source with addition of crust-derived components by oceanic subduction in an active continental margin setting^9,31,37^. The regional sedimentary sequences of Sibao and Danzhou Group were deposited at 860–832 and 803–764 Ma and the regional unconformity between them sealed the Jiangnan Orogen and was constrained to be 832–803 Ma, consistent with the presence of 833–822 Ma syn-collisional S-type granites^15,38^. These data with geological observations indicate the development of the early Neoproterozoic continental arc—basin system along the Jiangnan Orogen. Wang et al.^39^ and Gan et al.^40^ reported Silurian (ca. 430 Ma) arc-like gabbros and volcanics along the Yunkai-Nanling Domain and Early Jurassic olivine gabbros (ca. 191 Ma) in the Eastern Nanling Range, which have been regarded as derivation of an ancient lithospheric source inherited from the Neoproterozoic metasomatized wedge. When recalculated to 130 Ma, the previously reported Neoproterozoic mafic rocks^9,41^ in the studied areas exhibit *ε*_Nd_(*t*) values of − 2.2 to − 14.5, covering the ranges of *ε*_Nd_(*t*) values of the investigated mafic rocks in this study (Fig. 5).

Thus, we conclude that, the source for the studied mafic rocks was introduced by the subduction-derived components which is originally with high δ^18^O values via the Neoproterozoic subduction process along the western Qin-Hang belt.

## Methods

### Major, trace element and isotopic analyses

Major and trace element analyses were followed those methods described by Hou et al.^42^ and Qi et al.^43^, respectively. In brief, we crushed the rock samples to ~ 200 mesh powder in an agate mill. The analyses were carried out at the National Research Center of GeoAnalysis, Chinese Academy of Geological Sciences, Beijing. Major elements were analyzed by wet chemistry and X-ray fluorescence spectrometry (XRF) with analytical uncertainties ranging from 0.5 to 1.5%. Two standards (granite GSR-1, basalt GSR-3) were used to monitor the analytical quality. Trace elements were determined with a POEMS inductively coupled plasma mass spectrometry (ICP-MS). Analytical uncertainties are 10% for elements with abundances < 10 ppm, and around 5% for those > 10 ppm.

Sm–Nd isotope analyses of whole rock samples followed those procedures described by Chen et al.^44^. The whole rock samples (MD-09, QMS-14, WD-1 and WD-4) were determined by a Finnigan Triton TI thermal ionization mass spectrometer at Tianjin Institute of Geology and Mineral Resources, Tianjin. Procedural blanks yield concentration of < 200 pg for Sm and Nd, and mass fractionation corrections for Nd isotopic ratios were based on ^146^Nd/^144^Nd = 0.7219. The BCR-2 standard was run regularly and yielded a ^143^Nd/^144^Nd of 0.512642 ± 56 (2σ, n = 2). Isotopic analyses of other samples were performed using a Neptune Plus (Thermo Fisher Scientific, MA, USA) multi-collection mass spectrometer equipped with nine Faraday cups and eight ion counters at the Guangzhou Institute of Geochemistry, Chinese Academy of Sciences, Guangzhou. The BHVO-2 standard was run regularly and yielded a ^143^Nd/^144^Nd of 0.512957 ± 47 (2σ, n = 2). Measured ^143^Nd/^144^Nd ratios were normalized to ^146^Nd/^144^Nd = 0.7219.

### Zircon U–Pb dating and Hf–O isotope analysis

Zircon grains were separated from three mafic rocks using standard density and magnetic separation procedures and handpicked under a binocular microscope at the Rock and Minerals Experimental Testing Center of Xinhang Surveying and Mapping Institute, Langfang, Hebei Province. Zircon grains, together with zircon standards Plešovice and Qinghu were mounted together in epoxy discs and then polished to expose the longitudinal section of crystals in half for further analysis. All zircon grains were documented with transmitted and reflected light micrographs as well as CL images to reveal their internal structures. The CL images were performed using the JSM6510 scanning electron microscope attached with a GATAN Chroma CL detector housed at Beijing GeoAnalysis.

#### Zircon U–Pb dating by SIMS and LA-ICP-MS

Samples from Mandong area (MD-15) and Wende village (WD-1) were vacuum-coated with high purity gold prior to SIMS analysis. Measurements of elements U, Th, and Pb, were performed using a Cameca IMS-1280 SIMS at the Institute of Geology and Geophysics, Chinese Academy of Sciences (IGGCAS) in Beijing. The details of the analytical procedures have been described by Li et al.^45^. The O_2_^−**·**^ primary ion beam was accelerated at 13 kV, with an intensity of ca. 10 nA. The resulted ellipsoidal spot is about 20 × 30 μm in size. Positive secondary ions were extracted with a 10 kV potential. In the secondary ion beam optics, a 60 eV energy window was used, together with a mass resolution of ca. 5400 (at 10% peak height), to resolve lead isotopes from isobaric interferences. A single electron multiplier was used in ion-counting mode to measure secondary ion beam intensities by peak jumping mode. Each measurement consists of 7 cycles with the total analytical time of ca. 12 min. U–Th–Pb ratios were determined relative to the standard zircon Plešovice (^206^Pb/^238^U age of 337 Ma)^46^, and the absolute abundances were calibrated to the 91500 standard zircon (81.2 ppm U and 29 ppm Th)^47^. Pb/U calibration was performed in relation to standard zircon Plešovice and analyses of standards were interspersed with unknown grains. Measured compositions were corrected for common Pb using non-radiogenic ^204^Pb. An average of present-day crustal composition^48^ is used for the common Pb assuming that it is largely surface contamination introduced during sample preparation. Data reduction was carried out using the Isoplot/Ex v. 3.00 Program^49^. Uncertainties on individual analyses in data tables are reported at 1 sigma level; mean ages for pooled U/Pb analyses are quoted with 95% confidence interval.

U–Pb dating of zircons from Gaobang gabbro (GB2014-1) was performed by LA-ICP-MS at the Key Laboratory of Continental Collision and Plateau Uplift, Institute of Tibetan Plateau Research, Chinese Academy of Sciences in Beijing. A NewWave UP193FX Excimer laser coupled with an Agilent 7500a ICP-MS were used for determination of zircon U–Pb ages and trace element concentrations. The ablation system operated at a wavelength of 193 nm with a spot diameter of 35 µm. Each analysis consists of 15 s background acquisition, 40 s sample data acquisition, and a 45 s washout delay at the end. During the zircon analyses, the standard samples of zircon Plešovice and glass NIST SRM 612 were analyzed first, followed by 8 unknown sample analyses. The standard zircon Plešovice was used for correction of isotope fractionation. Common Pb was corrected following the method proposed by Andersen^50^. U–Pb ages were calculated using the GLITTER 4.0^51^ and age plots were carried out using the Isoplot 3.0 program^49^.

#### Zircon Lu–Hf isotope analysis

In situ Lu–Hf isotopic compositions were measured in previously dated zircon grains at the same domain. Zircon Hf isotope analysis was carried out on a Neptune multi-collector ICP-MS equipped with New Wave UP 213 laser-ablation system at the Institute of Mineral Resources, Chinese Academy of Geological Sciences, Beijing. Instrumental conditions and analytical procedures were comprehensively described by Hou et al.^52^ and a summary is present here. A stationary spot was used for analyses, with a beam diameter of 55 μm. Helium was used as a carrier gas to transport the ablated samples mixed with Argon from the laser-ablation cell to the ICP-MS torch via a mixing chamber. The isobaric interference of ^176^Lu and ^176^Yb on ^176^Hf was corrected using ratios of ^176^Lu/^175^Lu = 0.02658 and ^176^Yb/^173^Yb = 0.796218 proposed by Chu et al.^53^. For instrumental mass bias correction, Yb isotope ratios were normalized to ^172^Yb/^173^Yb = 1.35274^53^ and Hf isotope ratios to ^179^Hf/^177^Hf = 0.7325 using an exponential law^54^. Our routine run of the zircon standard GJ1 gave a weighted mean ^176^Hf/^177^Hf ratio of 0.281979 ± 0.000009 (2σ, n = 7), which is in good agreement with the reported value (0.282015 ± 0.000019)^55^.

#### Zircon oxygen isotope analysis by SIMS

Zircon oxygen isotopes were analyzed using the same Cameca IMS 1280 ion microprobe at IGGCAS, following the analytical procedures of Li et al.^56^. A focused Cs^+^ primary ion beam was accelerated at 10 kV with an intensity of ca. 2 nA. The analyzed spot is ca. 10 μm in diameter. In order to compensate for sample charging, a normal incidence electron flood gun was used with homogeneous electron density over a 100 μm elliptical area. Oxygen isotopes were measured using multi-collection mode on two off-axis Faraday cups (FC). The instrumental mass fractionation (IMF) during the analysis was corrected using the Penglai zircon standard with a δ^18^O value of 5.25‰^57^. Repeated analyzes of an in-house standard Qinghu zircon during the courses of sample analysis yielded mean δ^18^O value of 5.30 ± 0.23‰ (2σ, n = 8), which is identical within errors to the reported value (5.39 ± 0.22‰)^58^.

We state that our research did not carry out experiments involving human participants including the use of tissue samples. Our manuscript also did not contain information or images that could lead to identification of a study participant.

## Supplementary Information

Supplementary Information.
