# Supplementary material for: New identification and significance of Early Cretaceous mafic rocks in the interior South China Block
Source: Sci Rep. 2021 May 31;11:11396. doi: 10.1038/s41598-021-91045-1 (PMC8166846; doi:10.1038/s41598-021-91045-1)
Supplement: Supplementary file 1 — Supplementary Information. [file 41598_2021_91045_MOESM1_ESM.docx]

Supplementary materials

**New identification and significance of Early Cretaceous mafic rocks in the interior South China Block**

Hui-Min Su^1^, Shao-Yong Jiang^1^*, Jia-Bin Shao^1^, Dong-Yang Zhang^1^, Xiang-Ke Wu^2^, Xi-Qiang Huang^2^

^1^ State Key Laboratory of Geological Processes and Mineral Resources, Collaborative Innovation Center for Exploration of Strategic Mineral Resources, School of Earth Resources, China University of Geosciences, Wuhan 430074, China

^2^ Guangxi Institute of Geological Survey, Guangxi Bureau of Geology and Mineral Prospecting and Exploitation, Nanning 530023, China

*Correspondence to shyjiang@cug.edu.cn

**Supplementary Figure S1** Representative field photographs of the studied mafic rocks in Baotan area, northern Guangxi. (a, b) the Mandong gabbro and diabase intruded into the Neoproterozoic metasandstone; (c) the Mandong gabbro is in fault contact with the Neoproterozoic mineralized pyroxenite; (d) the Gaobang gabbro intruded into the Neoproterozoic metasandstone.


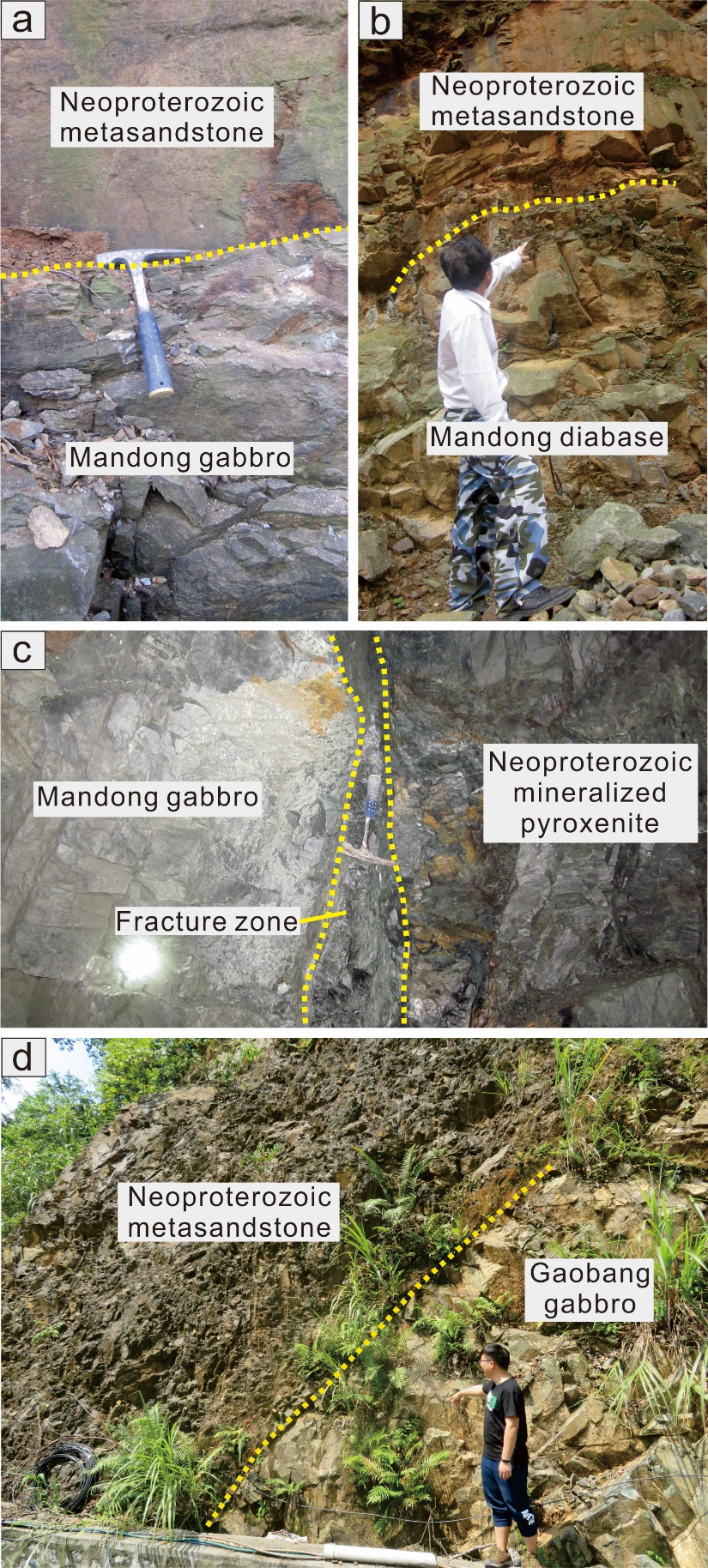


**Supplementary Figure S2** Photomicrographs of the studied mafic rocks in the Baotan area, northern Guangxi. (a, b) Mandong and Gaobang gabbros showing gabbro texture; (c, d) Wende diabases showing porphyritic texture. Mineral abbreviations: Cpx, clinopyroxene; Pl, plagioclase; Am, amphibole; Bi, biotite; Q, quartz.


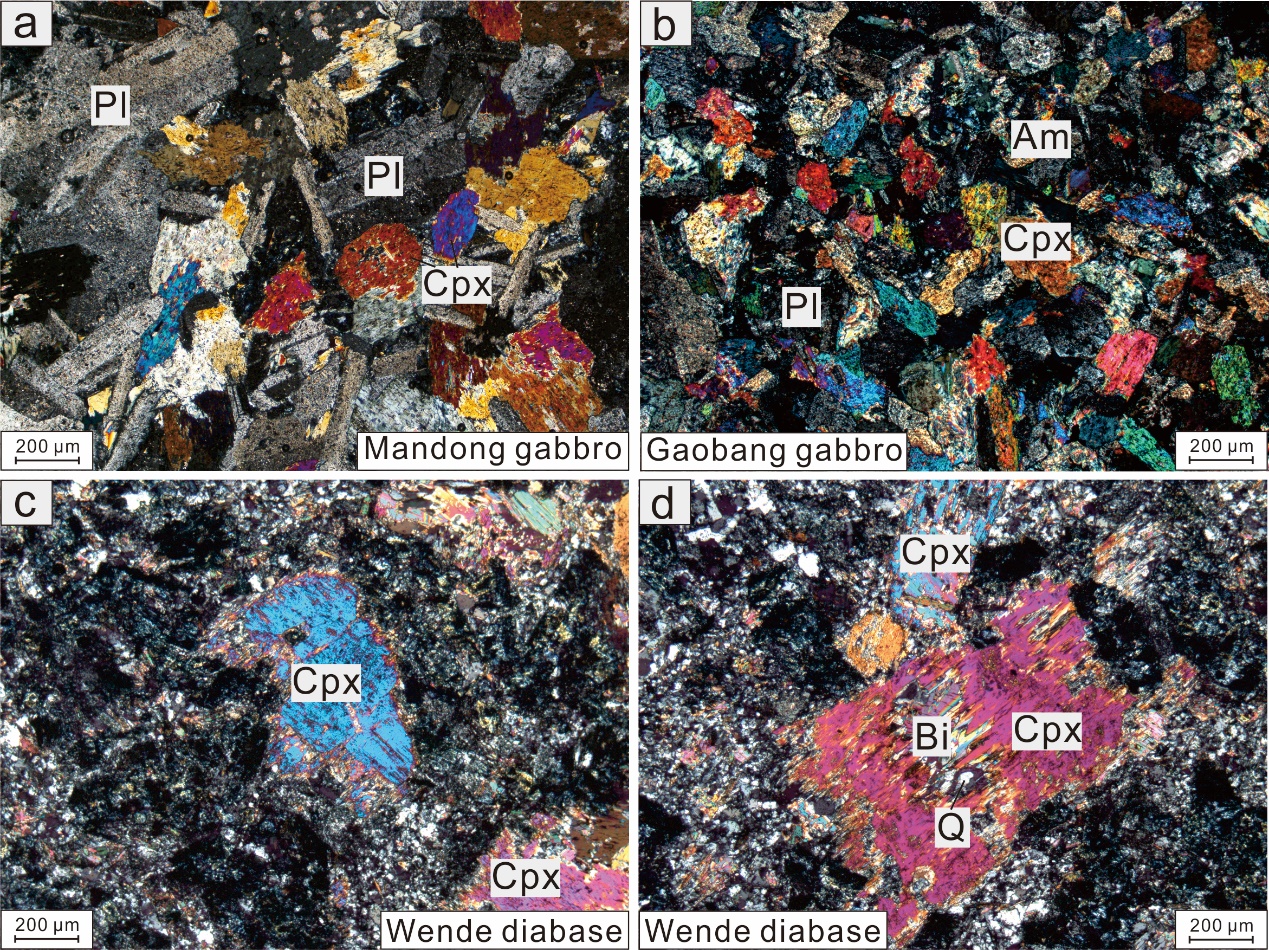


**Supplementary Table S1** Zircon U-Pb SIMS and LA-ICP-MS data for the mafic rocks in the Baotan area.

| **No.** | **Th**  **(ppm)** | **U**  **(ppm)** | **Th/U** | **Corrected ratios** | | | | | | **Corrected ages** | | | | | | **Concordance** |
| --- | --- | --- | --- | --- | --- | --- | --- | --- | --- | --- | --- | --- | --- | --- | --- | --- |
|  |  |  |  | **^207^Pb/^206^Pb** | **1σ** | **^207^Pb/^235^U** | **1σ** | **^206^Pb/^238^U** | **1σ** | **^207^Pb/^206^Pb** | **1σ** | **^207^Pb/^235^U** | **1σ** | **^206^Pb/^238^U** | **1σ** |  |
| **Mandong diabase (MD-15)** | | | | | | | | | | | | | | | | |
| 1 | 76 | 98 | 0.77 | 0.04470 | 8.29 | 0.12679 | 8.47 | 0.02057 | 1.73 | -72 | 191 | 121 | 10 | 131 | 2 | 92 |
| 2 | 62 | 98 | 0.63 | 0.04640 | 9.10 | 0.12586 | 9.25 | 0.01967 | 1.62 | 19 | 205 | 120 | 11 | 126 | 2 | 96 |
| 3 | 559 | 380 | 1.47 | 0.14571 | 0.83 | 8.37235 | 1.72 | 0.41672 | 1.50 | 2296 | 14 | 2272 | 16 | 2246 | 29 | 102 |
| 4 | 57 | 95 | 0.60 | 0.05301 | 3.21 | 0.14852 | 3.72 | 0.02032 | 1.88 | 329 | 71 | 141 | 5 | 130 | 2 | 108 |
| 5 | 136 | 175 | 0.78 | 0.04772 | 5.04 | 0.13315 | 5.27 | 0.02024 | 1.56 | 85 | 115 | 127 | 6 | 129 | 2 | 98 |
| 6 | 101 | 63 | 1.60 | 0.06294 | 2.23 | 1.08232 | 2.70 | 0.12472 | 1.52 | 706 | 47 | 745 | 14 | 758 | 11 | 98 |
| 7 | 272 | 314 | 0.87 | 0.05018 | 1.76 | 0.14201 | 2.32 | 0.02053 | 1.50 | 203 | 40 | 135 | 3 | 131 | 2 | 103 |
| 8 | 193 | 357 | 0.54 | 0.05584 | 1.23 | 0.56231 | 1.95 | 0.07303 | 1.51 | 446 | 27 | 453 | 7 | 454 | 7 | 100 |
| *9* | *466* | *493* | *0.95* | *0.04804* | *2.63* | *0.08414* | *3.12* | *0.01270* | *1.68* | *101* | *61* | *82* | *2* | *81* | *1* | *101* |
| 10 | 187 | 236 | 0.79 | 0.05055 | 2.17 | 0.14605 | 2.74 | 0.02095 | 1.67 | 220 | 49 | 138 | 4 | 134 | 2 | 104 |
| 11 | 273 | 484 | 0.57 | 0.05511 | 1.14 | 0.51641 | 1.89 | 0.06796 | 1.51 | 417 | 25 | 423 | 7 | 424 | 6 | 100 |
| 12 | 246 | 247 | 0.99 | 0.05030 | 2.67 | 0.14326 | 3.08 | 0.02066 | 1.54 | 209 | 61 | 136 | 4 | 132 | 2 | 103 |
| 13 | 427 | 782 | 0.55 | 0.05514 | 0.62 | 0.49084 | 1.63 | 0.06457 | 1.51 | 418 | 14 | 405 | 5 | 403 | 6 | 101 |
| 14 | 175 | 194 | 0.90 | 0.04747 | 5.02 | 0.13691 | 5.26 | 0.02092 | 1.55 | 73 | 115 | 130 | 6 | 133 | 2 | 98 |
| 15 | 531 | 802 | 0.66 | 0.04945 | 1.24 | 0.14149 | 1.96 | 0.02075 | 1.51 | 169 | 29 | 134 | 2 | 132 | 2 | 101 |
| **Wende diabase (WD-1)** | | | | | | | | | | | | | | | | |
| 1 | 77 | 105 | 0.73 | 0.05033 | 4.95 | 0.14288 | 5.24 | 0.02059 | 1.73 | 210 | 111 | 136 | 7 | 131 | 2 | 103 |
| 2 | 174 | 220 | 0.79 | 0.04825 | 3.11 | 0.13705 | 3.47 | 0.02060 | 1.54 | 112 | 72 | 130 | 4 | 131 | 2 | 99 |
| 3 | 59 | 104 | 0.57 | 0.04981 | 3.16 | 0.14217 | 3.68 | 0.02070 | 1.89 | 186 | 72 | 135 | 5 | 132 | 2 | 102 |
| *4* | *127* | *152* | *0.83* | *0.04120* | *11.84* | *0.11360* | *11.94* | *0.02000* | *1.56* | *-275* | *275* | *109* | *12* | *128* | *2* | ***86*** |
| 5 | 1226 | 2015 | 0.61 | 0.04830 | 0.84 | 0.14269 | 1.72 | 0.02143 | 1.50 | 114 | 20 | 135 | 2 | 137 | 2 | 99 |
| 6 | 300 | 312 | 0.96 | 0.04860 | 1.99 | 0.13722 | 2.56 | 0.02048 | 1.62 | 128 | 46 | 131 | 3 | 131 | 2 | 100 |
| 7 | 186 | 214 | 0.87 | 0.04717 | 3.87 | 0.13286 | 4.21 | 0.02043 | 1.66 | 58 | 90 | 127 | 5 | 130 | 2 | 97 |

**Table 1 (continued)**

| **No.** | **Th**  **(ppm)** | **U**  **(ppm)** | **Th/U** | **Corrected ratios** | | | | | | **Corrected ages** | | | | | | **Concordance** |
| --- | --- | --- | --- | --- | --- | --- | --- | --- | --- | --- | --- | --- | --- | --- | --- | --- |
|  |  |  |  | **^207^Pb/^206^Pb** | **1σ** | **^207^Pb/^235^U** | **1σ** | **^206^Pb/^238^U** | **1σ** | **^207^Pb/^206^Pb** | **1σ** | **^207^Pb/^235^U** | **1σ** | **^206^Pb/^238^U** | **1σ** |  |
| 8 | 175 | 201 | 0.87 | 0.04975 | 2.88 | 0.14073 | 3.26 | 0.02052 | 1.53 | 183 | 66 | 134 | 4 | 131 | 2 | 102 |
| 9 | 166 | 195 | 0.85 | 0.05016 | 2.27 | 0.14296 | 2.74 | 0.02067 | 1.53 | 203 | 52 | 136 | 3 | 132 | 2 | 103 |
| 10 | 125 | 156 | 0.80 | 0.04810 | 3.46 | 0.13364 | 3.82 | 0.02015 | 1.61 | 104 | 80 | 127 | 5 | 129 | 2 | 99 |
| 11 | 79 | 139 | 0.56 | 0.04477 | 7.29 | 0.12169 | 7.48 | 0.01971 | 1.63 | -68 | 169 | 117 | 8 | 126 | 2 | 93 |
| 12 | 189 | 224 | 0.84 | 0.04907 | 2.84 | 0.14086 | 3.24 | 0.02082 | 1.56 | 151 | 65 | 134 | 4 | 133 | 2 | 101 |
| 13 | 531 | 1170 | 0.45 | 0.04837 | 3.51 | 0.16316 | 3.82 | 0.02446 | 1.51 | 117 | 81 | 153 | 5 | 156 | 2 | 98 |
| 14 | 413 | 851 | 0.49 | 0.05430 | 0.60 | 0.47228 | 1.62 | 0.06308 | 1.50 | 384 | 13 | 393 | 5 | 394 | 6 | 100 |
| 15 | 2387 | 1499 | 1.59 | 0.05667 | 4.70 | 0.40112 | 4.93 | 0.05134 | 1.51 | 479 | 101 | 342 | 14 | 323 | 5 | 106 |
| **Gaobang gabbro (GB2014-1)** | | | | | | | | | | | | | | | | |
| 1 | 112 | 670 | 0.17 | 0.05393 | 0.00276 | 0.25954 | 0.01286 | 0.03491 | 0.00042 | 368 | 90 | 234 | 10 | 221 | 3 | 106 |
| 2 | 272 | 3162 | 0.09 | 0.05003 | 0.00115 | 0.14935 | 0.00311 | 0.02166 | 0.00015 | 196 | 35 | 141 | 3 | 138 | 1 | 102 |
| 3 | 654 | 1422 | 0.46 | 0.05186 | 0.00155 | 0.27420 | 0.00772 | 0.03836 | 0.00032 | 279 | 49 | 246 | 6 | 243 | 2 | 101 |
| 4 | 143 | 352 | 0.41 | 0.06564 | 0.00139 | 1.18498 | 0.02258 | 0.13096 | 0.00100 | 795 | 27 | 794 | 10 | 793 | 6 | 100 |
| 5 | 190 | 390 | 0.49 | 0.10253 | 0.00207 | 4.16863 | 0.07622 | 0.29498 | 0.00273 | 1670 | 20 | 1668 | 15 | 1666 | 14 | 100 |
| 6 | 115 | 754 | 0.15 | 0.16277 | 0.00193 | 10.52417 | 0.08726 | 0.46908 | 0.00308 | 2485 | 6 | 2482 | 8 | 2480 | 14 | 100 |
| 7 | 417 | 371 | 1.12 | 0.04925 | 0.00551 | 0.14458 | 0.01592 | 0.02130 | 0.00043 | 160 | 209 | 137 | 14 | 136 | 3 | 101 |
| 8 | 335 | 733 | 0.46 | 0.05021 | 0.00241 | 0.17943 | 0.00831 | 0.02593 | 0.00029 | 205 | 87 | 168 | 7 | 165 | 2 | 102 |
| *9* | *133* | *439* | *0.30* | *0.14933* | *0.00215* | *7.18379* | *0.08871* | *0.34890* | *0.00259* | *2338* | *25* | *2134* | *11* | *1929* | *12* | *121* |
| 10 | 347 | 651 | 0.53 | 0.05005 | 0.00221 | 0.19680 | 0.00840 | 0.02853 | 0.00029 | 197 | 80 | 182 | 7 | 181 | 2 | 101 |
| 11 | 279 | 549 | 0.51 | 0.05013 | 0.00227 | 0.17652 | 0.00773 | 0.02555 | 0.00027 | 201 | 82 | 165 | 7 | 163 | 2 | 101 |
| 12 | 193 | 429 | 0.45 | 0.05099 | 0.00261 | 0.17755 | 0.00881 | 0.02527 | 0.00029 | 240 | 94 | 166 | 8 | 161 | 2 | 103 |
| *13* | *346* | *398* | *0.87* | *0.05996* | *0.00418* | *0.31218* | *0.02141* | *0.03776* | *0.00046* | *602* | *155* | *276* | *17* | *239* | *3* | *115* |
| 14 | 467 | 1205 | 0.39 | 0.04866 | 0.00727 | 0.15760 | 0.02344 | 0.02349 | 0.00034 | 132 | 293 | 149 | 21 | 150 | 2 | 99 |
| 15 | 335 | 577 | 0.58 | 0.04605 | 0.00230 | 0.17770 | 0.00867 | 0.02799 | 0.00029 |  | 108 | 166 | 7 | 178 | 2 | 93 |
| 16 | 144 | 224 | 0.64 | 0.17113 | 0.00257 | 11.56892 | 0.14838 | 0.49048 | 0.00432 | 2569 | 11 | 2570 | 12 | 2573 | 19 | 100 |

**Table 1 (continued)**

| **No.** | **Th**  **(ppm)** | **U**  **(ppm)** | **Th/U** | **Corrected ratios** | | | | | | **Corrected ages** | | | | | | **Concordance** |
| --- | --- | --- | --- | --- | --- | --- | --- | --- | --- | --- | --- | --- | --- | --- | --- | --- |
|  |  |  |  | **^207^Pb/^206^Pb** | **1σ** | **^207^Pb/^235^U** | **1σ** | **^206^Pb/^238^U** | **1σ** | **^207^Pb/^206^Pb** | **1σ** | **^207^Pb/^235^U** | **1σ** | **^206^Pb/^238^U** | **1σ** |  |
| 17 | 98 | 151 | 0.65 | 0.18979 | 0.00265 | 13.85329 | 0.16024 | 0.52957 | 0.00444 | 2740 | 9 | 2740 | 11 | 2740 | 19 | 100 |
| 18 | 99 | 187 | 0.53 | 0.16247 | 0.00228 | 10.58753 | 0.12182 | 0.47280 | 0.00379 | 2482 | 10 | 2488 | 11 | 2496 | 17 | 99 |
| 19 | 42 | 100 | 0.42 | 0.17104 | 0.00259 | 11.60173 | 0.15104 | 0.49213 | 0.00437 | 2568 | 11 | 2573 | 12 | 2580 | 19 | 100 |
| 20 | 38 | 82 | 0.46 | 0.12124 | 0.00214 | 6.02093 | 0.09408 | 0.36029 | 0.00318 | 1975 | 16 | 1979 | 14 | 1984 | 15 | 100 |
| 21 | 82 | 309 | 0.27 | 0.06819 | 0.00139 | 1.33610 | 0.02442 | 0.14215 | 0.00107 | 874 | 25 | 862 | 11 | 857 | 6 | 101 |
| 22 | 29 | 115 | 0.25 | 0.11601 | 0.00187 | 5.50982 | 0.07591 | 0.34457 | 0.00276 | 1896 | 14 | 1902 | 12 | 1909 | 13 | 99 |
| *23* | *244* | *455* | *0.54* | *0.11132* | *0.00164* | *2.40342* | *0.02852* | *0.15664* | *0.00108* | *1821* | *12* | *1244* | *9* | *938* | *6* | *133* |
| 24 | 406 | 382 | 1.06 | 0.16825 | 0.00200 | 11.23557 | 0.09543 | 0.48449 | 0.00325 | 2540 | 7 | 2543 | 8 | 2547 | 14 | 100 |
| 25 | 13 | 22 | 0.61 | 0.16689 | 0.00451 | 11.06699 | 0.29550 | 0.48111 | 0.00758 | 2527 | 25 | 2529 | 25 | 2532 | 33 | 100 |
| 26 | 58 | 195 | 0.30 | 0.16896 | 0.00245 | 11.33947 | 0.13890 | 0.48689 | 0.00413 | 2547 | 10 | 2551 | 11 | 2557 | 18 | 100 |
| 27 | 10 | 27 | 0.37 | 0.17605 | 0.00377 | 12.09733 | 0.24932 | 0.49851 | 0.00644 | 2616 | 18 | 2612 | 19 | 2607 | 28 | 100 |
| 28 | 135 | 312 | 0.43 | 0.17018 | 0.00224 | 11.38301 | 0.11880 | 0.48525 | 0.00369 | 2559 | 8 | 2555 | 10 | 2550 | 16 | 100 |
| 29 | 286 | 539 | 0.53 | 0.05058 | 0.00211 | 0.17086 | 0.00688 | 0.02451 | 0.00024 | 222 | 75 | 160 | 6 | 156 | 2 | 103 |
| 30 | 284 | 341 | 0.83 | 0.05110 | 0.00302 | 0.25910 | 0.01492 | 0.03678 | 0.00048 | 245 | 109 | 234 | 12 | 233 | 3 | 100 |
| 31 | 123 | 671 | 0.18 | 0.06836 | 0.00137 | 1.24199 | 0.02228 | 0.13181 | 0.00100 | 879 | 25 | 820 | 10 | 798 | 6 | 103 |
| 32 | 221 | 321 | 0.69 | 0.04892 | 0.00736 | 0.14326 | 0.02118 | 0.02124 | 0.00061 | 144 | 260 | 136 | 19 | 135 | 4 | 101 |
| 33 | 526 | 783 | 0.67 | 0.05027 | 0.00129 | 0.28174 | 0.00672 | 0.04066 | 0.00030 | 207 | 42 | 252 | 5 | 257 | 2 | 98 |
| 34 | 76 | 1207 | 0.06 | 0.05348 | 0.00170 | 0.15337 | 0.00461 | 0.02080 | 0.00017 | 349 | 53 | 145 | 4 | 133 | 1 | 109 |
| 35 | 510 | 890 | 0.57 | 0.05000 | 0.00182 | 0.14569 | 0.00508 | 0.02114 | 0.00018 | 195 | 65 | 138 | 5 | 135 | 1 | 102 |
| 36 | 657 | 1668 | 0.39 | 0.05265 | 0.00358 | 0.15195 | 0.01007 | 0.02093 | 0.00030 | 314 | 126 | 144 | 9 | 134 | 2 | 107 |
| 37 | 272 | 1189 | 0.23 | 0.04998 | 0.00171 | 0.14610 | 0.00476 | 0.02120 | 0.00018 | 194 | 60 | 138 | 4 | 135 | 1 | 102 |
| 38 | 1235 | 643 | 1.92 | 0.05228 | 0.00316 | 0.25262 | 0.01484 | 0.03505 | 0.00049 | 298 | 109 | 229 | 12 | 222 | 3 | 103 |
| 39 | 613 | 1474 | 0.42 | 0.04690 | 0.00186 | 0.13943 | 0.00531 | 0.02156 | 0.00020 | 44 | 65 | 133 | 5 | 138 | 1 | 96 |
| 40 | 137 | 931 | 0.15 | 0.05107 | 0.00231 | 0.14922 | 0.00651 | 0.02119 | 0.00022 | 244 | 82 | 141 | 6 | 135 | 1 | 104 |

**Supplementary Table S2** Major and trace element analyses of mafic rocks in the Baotan area.

| Sample | Mandong | | | |  | Wend |  | Gaobang | | | |
| --- | --- | --- | --- | --- | --- | --- | --- | --- | --- | --- | --- |
|  | Gabbros | | Diabase | | Diabase | | | Gabbros | | | |
|  | QMS-9 | MD-09 | QMS-13 | QMS-16 | WD-1 | WD-3 | WD-4 | GB-01 | GB-04 | GB-05 | GB-06 |
| Major elements (wt.%) | | | | | | | | | | | |
| SiO_2_ | 50.86 | 52.88 | 51.27 | 54.40 | 51.47 | 53.12 | 53.35 | 53.55 | 55.35 | 56.09 | 54.14 |
| TiO_2_ | 0.53 | 0.65 | 0.50 | 0.55 | 0.34 | 0.33 | 0.34 | 0.58 | 0.50 | 0.47 | 0.58 |
| Al_2_O_3_ | 16.74 | 12.34 | 14.41 | 12.55 | 16.86 | 17.20 | 16.92 | 14.84 | 14.74 | 14.55 | 14.75 |
| FeOtot | 7.46 | 8.94 | 7.70 | 7.26 | 7.84 | 7.16 | 7.34 | 8.52 | 8.85 | 8.58 | 8.34 |
| MnO | 0.13 | 0.19 | 0.15 | 0.13 | 0.17 | 0.16 | 0.15 | 0.18 | 0.16 | 0.17 | 0.17 |
| MgO | 7.83 | 9.81 | 8.73 | 8.64 | 7.72 | 7.22 | 7.51 | 6.69 | 4.99 | 5.30 | 6.61 |
| CaO | 8.76 | 6.16 | 9.27 | 8.28 | 9.46 | 8.64 | 8.11 | 8.77 | 8.88 | 8.64 | 9.45 |
| Na_2_O | 1.80 | 2.42 | 3.04 | 4.16 | 0.48 | 0.83 | 0.86 | 3.16 | 2.55 | 2.13 | 2.39 |
| K_2_O | 2.20 | 1.90 | 0.81 | 0.42 | 1.39 | 1.57 | 2.03 | 1.01 | 0.75 | 1.25 | 1.11 |
| P_2_O_5_ | 0.06 | 0.07 | 0.05 | 0.05 | 0.05 | 0.05 | 0.06 | 0.06 | 0.07 | 0.07 | 0.06 |
| LOI | 2.43 | 3.41 | 2.58 | 2.07 | 2.91 | 2.88 | 2.87 | 2.03 | 1.50 | 1.37 | 1.64 |
| Total | 99.03 | 98.97 | 98.68 | 98.65 | 98.93 | 99.35 | 99.76 | 99.69 | 98.62 | 98.85 | 99.51 |
| Mg^#^ | 0.65 | 0.66 | 0.67 | 0.68 | 0.64 | 0.64 | 0.65 | 0.58 | 0.50 | 0.52 | 0.59 |
| Trace elements (ppm) | | | | | | | | | | | |
| Sc | 32.3 | 36.0 | 41.7 | 44.6 | 31.0 | 33.4 | 33.3 | 43.5 | 44.3 | 44.8 | 43.2 |
| V | 182 | 184 | 212 | 208 | 181 | 183 | 181 | 186 | 231 | 188 | 223 |
| Cr | 319 | 635 | 135 | 125 | 264 | 301 | 312 | 177 | 144 | 193 | 135 |
| Co | 40.4 | 48.3 | 42.0 | 47.4 | 27.5 | 27.5 | 28.5 | 30.9 | 35.6 | 30.4 | 36.7 |
| Ni | 73.3 | 125 | 54.2 | 55.8 | 8.84 | 9.35 | 9.47 | 5.98 | 63.0 | 6.32 | 61.3 |
| Cu | 63.0 | 186 | 19.6 | 84.0 | 0.62 | 0.18 | 111 | 4.90 | 25.2 | 3.28 | 60.9 |
| Zn | 60.7 | 83.9 | 67.9 | 54.4 | 90.0 | 81.8 | 82.0 | 80.3 | 79.8 | 81.0 | 77.7 |
| Ga | 15.2 | 13.0 | 14.8 | 10.0 | 17.1 | 15.6 | 15.3 | 15.9 | 14.7 | 15.1 | 14.6 |
| Rb | 145 | 117 | 48.5 | 22.4 | 75.8 | 90.4 | 127 | 30.0 | 51.4 | 56.9 | 46.1 |
| Sr | 126 | 103 | 151 | 66.0 | 182 | 164 | 132 | 100 | 117 | 102 | 123 |
| Y | 13.9 | 19.4 | 14.1 | 14.3 | 14.1 | 14.1 | 14.1 | 22.2 | 18.3 | 19.7 | 18.4 |
| Zr | 62.1 | 67.0 | 55.6 | 62.6 | 67.7 | 68.0 | 68.2 | 93.7 | 64.6 | 87.1 | 62.7 |
| Nb | 3.76 | 4.65 | 3.36 | 3.80 | 4.91 | 4.85 | 4.88 | 6.94 | 3.98 | 6.79 | 3.94 |
| Ba | 207 | 368 | 144 | 50.8 | 146 | 188 | 229 | 137 | 136 | 191 | 218 |
| La | 9.41 | 9.40 | 7.89 | 8.46 | 11.9 | 12.0 | 12.0 | 20.3 | 11.3 | 18.0 | 11.4 |
| Ce | 19.9 | 19.3 | 17.0 | 18.5 | 24.6 | 24.9 | 24.3 | 36.4 | 20.9 | 33.9 | 20.8 |
| Pr | 2.45 | 2.32 | 2.16 | 2.30 | 2.93 | 3.00 | 2.91 | 3.95 | 2.41 | 3.47 | 2.30 |
| Nd | 10.4 | 10.2 | 9.51 | 10.1 | 11.7 | 12.2 | 11.9 | 15.7 | 10.2 | 14.7 | 9.50 |
| Sm | 2.21 | 2.84 | 2.01 | 2.13 | 2.16 | 2.24 | 2.15 | 3.57 | 2.60 | 3.22 | 2.54 |
| Eu | 0.64 | 0.69 | 0.65 | 0.55 | 0.71 | 0.61 | 0.55 | 0.88 | 0.71 | 0.79 | 0.7 |
| Gd | 2.38 | 3.19 | 2.53 | 2.59 | 2.29 | 2.20 | 2.29 | 3.87 | 3.11 | 3.48 | 2.92 |
| Tb | 0.40 | 0.55 | 0.42 | 0.42 | 0.39 | 0.40 | 0.38 | 0.65 | 0.52 | 0.58 | 0.51 |
| Dy | 2.71 | 3.50 | 2.63 | 2.81 | 2.52 | 2.59 | 2.53 | 4.06 | 3.35 | 3.63 | 3.32 |
| Ho | 0.55 | 0.76 | 0.56 | 0.59 | 0.54 | 0.56 | 0.54 | 0.87 | 0.72 | 0.75 | 0.69 |
| Er | 1.47 | 2.18 | 1.45 | 1.50 | 1.58 | 1.52 | 1.42 | 2.54 | 2.13 | 2.28 | 2.12 |
| Tm | 0.26 | 0.31 | 0.24 | 0.24 | 0.25 | 0.25 | 0.25 | 0.38 | 0.31 | 0.34 | 0.30 |
| Yb | 1.49 | 2.02 | 1.65 | 1.54 | 1.69 | 1.69 | 1.66 | 2.54 | 1.97 | 2.34 | 1.91 |
| Lu | 0.26 | 0.32 | 0.25 | 0.25 | 0.26 | 0.29 | 0.27 | 0.41 | 0.32 | 0.38 | 0.33 |
| Hf | 1.71 | 2.07 | 1.54 | 1.74 | 1.80 | 1.86 | 1.85 | 2.98 | 2.13 | 2.80 | 2.19 |
| Ta | 0.30 | 0.38 | 0.28 | 0.30 | 0.37 | 0.37 | 0.38 | 0.60 | 0.34 | 0.56 | 0.34 |
| Pb | 3.97 | 5.71 | 5.49 | 4.93 | 6.41 | 6.89 | 7.20 | 7.68 | 4.49 | 7.85 | 4.82 |
| Th | 3.64 | 4.21 | 3.15 | 3.63 | 5.35 | 5.34 | 5.35 | 8.41 | 3.97 | 7.45 | 4.07 |
| U | 0.74 | 0.86 | 0.70 | 0.85 | 1.14 | 1.16 | 1.14 | 1.74 | 0.72 | 1.55 | 0.74 |
| ΣREE | 54.5 | 57.6 | 49.0 | 52.0 | 63.5 | 64.5 | 63.2 | 96.1 | 60.6 | 87.9 | 59.3 |
| (La/Yb)_N_ | 4.53 | 3.34 | 3.43 | 3.94 | 5.05 | 5.09 | 5.19 | 5.73 | 4.11 | 5.52 | 4.28 |
| Eu/Eu^*^ | 0.85 | 0.70 | 0.88 | 0.72 | 0.98 | 0.84 | 0.76 | 0.72 | 0.76 | 0.72 | 0.79 |

Note: Mg^#^ is MgO/(MgO+FeOT) in molar proportions.

**Supplementary Table S3** Whole-rock Sm-Nd isotopic compositions of mafic rocks in the Baotan area.

| **Sample** | **Rock type** | **Age**  **(Ma)** | **Sm**  **(ppm)** | **Nd**  **(ppm)** | **^147^Sm/^144^Nd** | **^143^Nd/^144^Nd** | **(^143^Nd/^144^Nd)_i_** | **ε_Nd_(t)** | **T_DM_ (Ma)** |
| --- | --- | --- | --- | --- | --- | --- | --- | --- | --- |
| QMS-9 | Mandong gabbro | 131 | 2.21 | 10.4 | 0.1285 | 0.512005±4 | 0.51189 | -11.23 | 2045 |
| MD-09 |  | 131 | 2.84 | 10.2 | 0.1683 | 0.512125±2 | 0.51198 | -9.53 | 3425 |
| QMS-14 | Mandong diabase | 131 | 2.01 | 9.51 | 0.1278 | 0.512076±4 | 0.51197 | -9.82 | 1903 |
| QMS-16 |  | 131 | 2.13 | 10.1 | 0.1275 | 0.512036±3 | 0.51193 | -10.58 | 1967 |
| WD-1 | Wende diabase | 131 | 2.16 | 11.7 | 0.1116 | 0.511892±2 | 0.51180 | -13.14 | 1876 |
| WD-3 |  | 131 | 2.24 | 12.2 | 0.111 | 0.511865±4 | 0.51177 | -13.65 | 1904 |
| WD-4 |  | 131 | 2.15 | 11.9 | 0.1092 | 0.511894±8 | 0.51180 | -13.06 | 1830 |
| GB-01 | Gaobang gabbro | 135 | 3.57 | 15.7 | 0.1374 | 0.511856±3 | 0.51174 | -14.27 | 2578 |
| GB-05 |  | 135 | 3.22 | 14.7 | 0.1324 | 0.511858±4 | 0.51174 | -14.16 | 2417 |
| BHVO-2a | Standard sample |  |  |  |  | 0.512961±3 |  |  |  |
| BHVO-2b |  |  |  |  |  | 0.512954±3 |  |  |  |
| BCR-2a |  |  |  |  |  | 0.512647±6 |  |  |  |
| BCR-2b |  |  |  |  |  | 0.512638±5 |  |  |  |

**Supplementary Table S4** Zircon Hf and O isotopes for mafic rocks in the Baotan area.

| **No.** | **Age (Ma)** | **^176^Yb/^177^Hf** | **^176^Lu/^177^Hf** | **^176^Hf/^177^Hf** | **2σ** | **^176^Hf/^177^Hf _i_** | ***ε*_Hf_(t)** | **f_Lu/Hf_** | **T_DM1_ (Ma)** | **T_DM2_ (Ma)** | **δ^18^O** | **2σ** |
| --- | --- | --- | --- | --- | --- | --- | --- | --- | --- | --- | --- | --- |
|  | **MD-15 (Mandong diabase)** | | | | | | | | | | | |
| 1 | 131 | 0.062361 | 0.001921 | 0.282450 | 0.000024 | 0.282445 | -8.68 | -0.94 | 1162 | 1734 | 5.72 | 0.19 |
| 2 | 126 | 0.046437 | 0.001455 | 0.282501 | 0.000023 | 0.282497 | -6.97 | -0.96 | 1076 | 1621 | 7.81 | 0.30 |
| 4 | 130 | 0.030825 | 0.000995 | 0.282519 | 0.000022 | 0.282517 | -6.17 | -0.97 | 1036 | 1575 | 7.08 | 0.22 |
| 5 | 129 | 0.038178 | 0.001183 | 0.282428 | 0.000023 | 0.282425 | -9.45 | -0.96 | 1171 | 1781 | 7.40 | 0.25 |
| 7 | 131 | 0.033149 | 0.001091 | 0.282433 | 0.000025 | 0.282431 | -9.19 | -0.97 | 1160 | 1766 | 8.07 | 0.28 |
| 10 | 134 | 0.031612 | 0.001036 | 0.282492 | 0.000023 | 0.282490 | -7.04 | -0.97 | 1075 | 1633 | 7.32 | 0.30 |
| 12 | 132 | 0.069462 | 0.002125 | 0.282442 | 0.000020 | 0.282437 | -8.96 | -0.94 | 1180 | 1752 | 8.09 | 0.26 |
| 14 | 133 | 0.051485 | 0.001572 | 0.282480 | 0.000023 | 0.282476 | -7.55 | -0.95 | 1109 | 1664 | 7.32 | 0.21 |
| 15 | 132 | 0.060838 | 0.001928 | 0.282427 | 0.000015 | 0.282423 | -9.45 | -0.94 | 1195 | 1783 | 6.81 | 0.31 |
|  | **WD-1 (Wende diabase)** | | | | | | | | | | | |
| 1 | 131 | 0.049999 | 0.001568 | 0.282431 | 0.000021 | 0.282427 | -9.32 | -0.95 | 1178 | 1775 | 7.13 | 0.34 |
| 2 | 131 | 0.015570 | 0.000515 | 0.282473 | 0.000020 | 0.282471 | -7.75 | -0.98 | 1088 | 1676 | 7.20 | 0.30 |
| 3 | 132 | 0.015607 | 0.000508 | 0.282470 | 0.000019 | 0.282469 | -7.83 | -0.98 | 1092 | 1682 | 7.89 | 0.32 |
| 5 | 137 | 0.046712 | 0.001473 | 0.282458 | 0.000017 | 0.282454 | -8.24 | -0.96 | 1137 | 1710 | 7.12 | 0.33 |
| 6 | 131 | 0.076557 | 0.002374 | 0.282431 | 0.000021 | 0.282425 | -9.39 | -0.93 | 1204 | 1778 | 7.40 | 0.21 |
| 7 | 130 | 0.036666 | 0.001139 | 0.282514 | 0.000021 | 0.282511 | -6.36 | -0.97 | 1048 | 1587 | 7.31 | 0.21 |
| 8 | 131 | 0.068169 | 0.002131 | 0.282478 | 0.000022 | 0.282473 | -7.72 | -0.94 | 1128 | 1673 | 7.35 | 0.24 |
| 9 | 132 | 0.049317 | 0.001534 | 0.282478 | 0.000021 | 0.282475 | -7.62 | -0.95 | 1110 | 1668 | 7.03 | 0.34 |
| 10 | 129 | 0.032623 | 0.001017 | 0.282486 | 0.000020 | 0.282484 | -7.37 | -0.97 | 1083 | 1649 | 7.04 | 0.20 |
| 12 | 133 | 0.038631 | 0.001203 | 0.282496 | 0.000024 | 0.282493 | -6.96 | -0.96 | 1075 | 1627 | 7.25 | 0.23 |
| 14 | 394 | 0.048393 | 0.001571 | 0.282599 | 0.000018 | 0.282588 | 2.16 | -0.95 | 938 | 1248 | 8.48 | 0.20 |
